# Evaluating the Spectrum of Sleep Abnormalities in Patients With Primary Hypothyroidism

**DOI:** 10.7759/cureus.69855

**Published:** 2024-09-21

**Authors:** Himamshu Acharya, Sanjana Jayaraj Mangala, Pramila Kalra

**Affiliations:** 1 Endocrinology and Metabolism, MS Ramaiah Medical College, Bengaluru, IND

**Keywords:** osa, primary hypothyroidism, questionnaires, sleep dysfunction, subclinical hypothyroidism

## Abstract

Introduction and aim: Primary hypothyroidism is a common endocrine disorder. While thyroid dysfunction is recognized for impacting numerous bodily systems, the connection between thyroid disorders and sleep function remains unclear. Since sleep disorders seldom manifest as the sole presenting symptom of thyroid dysfunction, it is crucial to examine the interplay between thyroid function and sleep when implementing a comprehensive treatment approach for these individuals. This study aimed to assess sleep dysfunction in patients with primary hypothyroidism using validated questionnaires.

Method: This cross-sectional study included 81 participants attending the endocrinology outpatient department. The participants included those with both overt and subclinical primary hypothyroidism who were drug-naive or had a thyroid-stimulating hormone (TSH) level >4.5 µIU/L while on treatment. The study subjects were assessed based on various validated questionnaires for sleep disturbance.

Results: The study predominantly comprised 64 (79%) female participants. Overall, poor sleepers in subclinical and overt hypothyroidism were 66.7 and 60.8%. The Berlin Risk Score Questionnaire showed the occurrence of sleep apnea to be 26.7% and 27.5% in subclinical and overt groups, respectively. However, the occurrence of sleep dysfunction did not correlate with TSH values alone.

Conclusion: Our findings demonstrate that sleep dysfunction is prevalent in patients with both overt and subclinical primary hypothyroidism. Furthermore, the severity of sleep disruption appears independent of the degree of thyroid dysfunction.

## Introduction

Primary hypothyroidism manifests across a broad clinical spectrum, encompassing overt myxedema, end-organ impacts, multisystem failure, as well as asymptomatic or subclinical states. Hypothyroidism is a common endocrine disorder with a prevalence of 4-10% [1]. In a densely populated and developing nation such as India, the primary focus on health concerns lies in non-communicable diseases, given their significant contribution to the overall national disease burden [2].

Sleep, a fundamental pillar of human health, is essential for physical and cognitive restoration. Disruptions in sleep architecture can significantly impact well-being, including mood, energy levels, and even cardiovascular health. Extensive investigations into sleep-related disorders have revealed that insufficient or disrupted sleep can substantially affect one's health in various ways. The International Society of Sleep Disorders lists the 81 types of sleep disorders into eight major categories [3]. Insomnia and sleep-related breathing disorders are the two entities under assessment in this context.

Peripheral obstructive sleep apnea syndrome (OSAS) is a sleep disorder marked by extended periods of partial obstruction or occasional complete upper airway blockage, leading to regular sleep pattern disruptions. Accompanied by significant neurobehavioral and cardio-respiratory repercussions, obstructive sleep apnea syndrome (OSAS) can result in conditions such as excessive daytime sleepiness (EDS), growth impairment, academic challenges, behavioral issues, cor pulmonale, motor vehicle accidents, and, in extreme cases, sudden death. A study has estimated OSAS prevalence to be 3.6% in an Indian community-based sample, translating to over 36 million affected individuals when extrapolated to an overall population of over one billion in India, which carries substantive public health significance [4].

The prevalence of hypothyroidism in obstructive sleep apnea (OSA) is 1.5-11% [5], and up to 30-74% of symptomatic hypothyroid patients can develop OSA [6,7]. Early detection of OSA in hypothyroidism can improve quality of life and reduce morbidity due to OSA. Universal screening of OSA with polysomnography (PSG) in hypothyroidism is not recommended at present. This study can help screen for OSA, thus helping identify individuals requiring PSG.

While thyroid dysfunction is recognized for impacting numerous bodily systems, the connection between thyroid disorders and sleep function remains unclear. Since sleep disorders seldom manifest as the sole presenting symptom of thyroid dysfunction, it is crucial to examine the interplay between thyroid function and sleep when implementing a comprehensive treatment approach for individuals with such disorders.

## Materials and methods

This cross-sectional, single-center study was conducted at the Department of Endocrinology in a tertiary care hospital over nine months. The ethical clearance was obtained from the ethics committee. All patients aged >18 years diagnosed with primary hypothyroidism attending the endocrinology outpatient department were included in the study. The study subjects were assigned into two following groups: (1) subclinical hypothyroidism (thyroid-stimulating hormone {TSH} value of 4.5-10 µIU/mL) and (2) overt hypothyroidism (TSH value of ≥10 µIU/mL, irrespective of T4). This included both newly diagnosed patients and those with a TSH value >4.5 µIU/mL despite treatment. Patients with severe cognitive deficits, which impair their ability to answer the questionnaire, diagnosed with psychiatric disorders before the onset of hypothyroidism were excluded from the study. The participants were delivered questionnaires to assess various composite variables. The questionnaires used in this study are explained below.

Pittsburgh Sleep Quality Index (PSQI)

PSQI consists of 19 self-rated questions and five questions rated by the bed partner/roommate (if present). Only self-administered questions were included in the scoring. The 19 self-rated items were included to form seven component scores, each with a range of 0-3 points (appendix 1). In all cases, a score of 0 showed no difficulty, while a score of 3 indicated severe difficulty. The seven-component score is then added to get a combined score with a range of 0-21, 0 being no difficulty and 21 indicating severe difficulty. A PSQI score over 5 indicates poor sleep quality [8].

Berlin Sleep Questionnaire

It is a 10-item questionnaire with three following categories: snoring severity, excessive daytime sleepiness, and a history of high blood pressure or obesity (appendix 2). Patients were grouped into high risk if two of the three categories were positive (a score of 2 or more was considered positive in each category) or low risk based on their response to the individual items and their overall scores in the symptom category [9].

Epworth Sleepiness Score (ESS)

The Epworth Sleepiness Score is a self-administered questionnaire with eight questions (appendix 3). Respondents were asked to rate, on a four-point scale (0-3), their usual chances of dozing off or falling asleep while engaged in eight different activities. Most people occasionally engage in those activities, although not every day. The ESS Score (sum of eight item score, 0-3) ranges from 0 to 24. The higher the ESS, the greater the chances that a person's average sleep propensity in daily life (ASP) or daytime sleepiness [10]. A score of 0-5 was considered low normal daytime sleepiness, and 6-10 as high normal daytime sleepiness. A score ≥11 was regarded as excessive daytime sleepiness.

Hamilton Anxiety Rating Scale (HAM-A)

Created to gauge and quantify the severity of symptoms in individuals with anxiety neurosis, this instrument is a 21-item self-report questionnaire (appendix 4). Its design aimed to evaluate the frequency of anxiety symptoms experienced by individuals over one week. The assessment conducted by this test focuses on the following two key factors: cognitive and somatic symptoms [11]. Each item was scored on a scale of zero (not present) to four (severe), with a total score of 0-56, where seven indicates mild severity, 18-24 mild-to-moderate severity, and 25-30 moderate-to-severe.

Montreal Cognitive Assessment (MOCA)

The MoCA evaluates various cognitive domains, which include visuospatial/executive, naming, memory, attention, language, abstraction, delayed recall, and orientation (to time and place) (appendix 5). The assessment was condensed into a 30-point test and to be completed in approximately 10 minutes. A score of 26 or higher was regarded as within the normal range [12].

Patient Health Questionnaire-9

The Patient Health Questionnaire (PHQ) is a self-administered adaptation of the PRIME-MD diagnostic tool (New York City, NY: Pfizer) designed for common mental disorders (appendix 6). Its primary purpose is to facilitate criteria-based diagnoses of prevalent depressive and other cognitive disorders frequently encountered in primary care settings. PHQ-9 is a concise nine-item questionnaire. PHQ-9 scores of 1-4, 5-9, 10-14, 15-19, and >20 depict minimal, mild, moderate, moderately severe, and severe depression, respectively [13].

Apart from the questionnaires, various physical parameters were considered. Waist hip ratio (WHR) was measured to assess visceral obesity. Waist measurement was taken mid-way between the lowest rib and the iliac crest and the hip circumference was measured at the transtubercular plane. Neck circumference (NC) was measured with the head positioned in the Frankfurt line, between the mid-cervical spine and mid-anterior neck (below Adam’s apple in males) with the help of calibrated measuring tape. Mallampati scores were assessed with the patient in a seated position and protruding the tongue maximally with no phonation. Neck height ratio (NhtR) was obtained by dividing the neck circumference and height of the subject.

All the measurements were done by the same individuals and the average of the three measurements were taken. The questionnaires were administered orally by the same individual. Blood samples were collected for the assessment of TSH. TSH was measured by chemiluminescence immunoassay (CLIA) (assay range: 0.5-4.50 µIU/mL).

Statistical methods

Study Design: Descriptive Cross-Sectional Study

Sample size: The sample size was calculated considering the proportion of sleep dysfunction as 74% as per the study by Pancholi et al. [7]. The sample size was calculated with 5% absolute precision and 95% confidence level. Based on the previous hospital records, the approximate number of potentially eligible subjects attending the study setting during the data collection period was considered 100. Hence a finite population correction was applied for 100. As per the calculation mentioned above, the required sample size was 75. To account for a non-participation rate/loss to follow-up rate of about 5%, another four subjects will be added to the sample size. The final required sample size would be 79.

The descriptive statistics of sleep apnea, cognitive assessment, anxiety, and depression scores were analyzed and summarized in terms of mean and standard deviation. The independent t-test/Mann-Whitney U test was used to compare means. The p-value <0.05 was considered significant. All data were analyzed using IBM SPSS version 20 (Armonk, NY: IBM Corp.).

## Results

The study included 81 subjects, of which 64 (79%) were females and 17 (21%) were males, with a mean age of 38.5±5 years. The demographic details of study participants are depicted in Table 1. The mean BMI of the study population was 26.65±5.22 kg/m^2^. All other parameters were similar among the study groups.

**Table 1:** Demographic details of study participants. P-value <0.05 was considered significant. No patients had Mallampati grade 4 severity. n: number in each group; N: total no of subjects; IQR: interquartile range

Variables	Overall (mean±SD)	Subclinical hypothyroidism (mean ±SD) N=30	Overt hypothyroidism (mean±SD) N=51	p-Value
Age (years)	38.5±13	40±13	38±13	0.465
Gender				
Male, n (%)	17 (21%)	7	10	0.69
Female, n (%)	64 (79%)	23	41	
TSH (µIU/mL)	33.53±56.6 (median: 13.05, IQR 7.82-25.19)	7.097±1.33	49.08±66.88 (median: 35, IQR:13.49-50.9)	-
BMI (kg/m^2^)	26.65±5.22	27.66±5.49	26.06±5.03	0.183
Neck circumference (cm)	34.88±3.74	35.6±3.7	34.5±3.7	0.209
Waist-to-hip ratio	0.92±0.07	0.93±0.08	0.91±0.07	0.181
Neck-to-height ratio	0.22±0.019	0.23±0.02	0.22±0.02	0.063
Mallampati score				
Grade 1 (n)	13	3	10	0.443
Grade 2 (n)	50	20	30	
Grade 3 (n)	18	7	11	

Sleep assessment

Two-thirds of the study population, i.e., 62.9%, had a PSQI score of ≥6, indicating poor sleep quality, of which, 20 (66.7%) were found in subclinical hypothyroidism and 30 (60.8%) constituted overt hypothyroidism (p=0.59). Approximately 27% had a high risk for sleep apnea among both subclinical and overt hypothyroidism (p=0.93). Thereby signifying equal occurrence of sleep apnea and poor sleep quality in both subclinical and overt hypothyroidism (Table 2).

**Table 2:** PSQI and Berlin Risk Score among the study population. P-value <0.05 was considered significant. PSQI: Pittsburgh Sleep Quality Index

Variables	Overall (n)	Subclinical hypothyroidism, n (%)	Overt hypothyroidism, n (%)	p-Value
PSQI (0-5)	30	10 (33.3)	20 (39.2)	0.599
PSQI ≥6	51	20 (66.7)	31 (60.8)	
Berlin Risk Score for sleep apnea low risk	59	22 (73.3)	37 (72.5)	0.939
Berlin Risk Score for sleep apnea high risk	22	8 (26.7)	14 (25.7)	

Further statistical analysis showed that a higher BMI had a higher risk of OSA as per the Berlin risk categorization. On analysis of Berlin risk categorization with neck-height ratio and collar circumference, a higher neck-height ratio and greater collar circumference were associated with a higher risk of OSA in females. However, the same parameters showed no statistically significant relation with male gender (Table 3).

**Table 3:** Berlin risk categorization and association of physical parameters for OSA assessment. *The 75th percentile of the data of collar circumference for males and females was taken as the cut-off. **The 75th percentile of the data of the neck-height ratio for males and females was used as the cut-off. P-value <0.05 was considered significant. OSA: obstructive sleep apnea

Parameters	Berlin risk categorization low-risk, n (%)	Berlin risk categorization high risk, n (%)	p-Value
BMI (kg/m^2^) ≤21	8 (14%)	1 (4.2%)	0.00
BMI (kg/m^2^) 21-23.9	12 (21.1%)	1 (4.2%)	
BMI (kg/m^2^) >24	37 (64.9%)	22 (91.7%)	
Collar (males)* ≤47.5 cm	10 (71.4%)	3 (100%)	0.29
Collar (males)* >47.6 cm	4 (28.6%)	0 (0)	
Collar (females)* ≤40 cm	37 (86%)	12 (57.1%)	0.01
Collar (females)* >40 cm	6 (14%)	9 (42.9%)	
Neck-height ratio (males)** ≤0.25	11(78.6%)	2 (66.7%)	0.6
Neck-height ratio (males)** >0.26	3 (21.4%)	1 (33.3%)	
Neck-height ratio (females)** ≤0.22	37 (86%)	8 (38.1%)	0.00
Neck-height ratio (females)** >0.23	6 (14%)	13 (61.9%)	

The results from the Epworth Sleepiness Score (Table 4) indicated that overall and within each study group, complaints of daytime sleepiness were minimal. In the overt hypothyroidism group, low-normal daytime sleepiness was observed in 37 participants (72.5%), which was relatively higher than the corresponding figure in the subclinical hypothyroidism group, where 17 individuals (56.7%) reported low-normal daytime sleepiness. This indicates that the range of thyroid dysfunction did not influence the presence of excessive daytime sleepiness. Both overt and subclinical hypothyroid patients are equally susceptible to experiencing these symptoms.

**Table 4:** Epworth Sleepiness Score assessment in study population. P-value <0.05 was taken as significant.

Epworth category	Overall (n=81)	Subclinical hypothyroidism, n (%)	Overt hypothyroidism (%)	p-Value
Low normal daytime sleepiness	54	17 (56.7%)	37 (72.5%)	0.412
Normal daytime sleepiness	14	8 (26.7%)	6 (11.8%)	
Mild excessive daytime sleepiness	9	4 (13.3%)	5 (9.8%)	
Moderate excessive daytime sleepiness	1	0	1 (2%)	
Severe excessive daytime sleepiness	3	1 (3.3%)	2 (3.9%)	

According to the PHQ-9 Questionnaire, there was a higher prevalence of moderate depression in the subclinical hypothyroidism group, with seven individuals (23.3%), compared to the overt hypothyroidism group, where it was 11.8%. Most subjects exhibited normal cognition in both study groups despite an underlying sleep disorder (Table 5). Out of the total 81 subjects, only seven individuals demonstrated cognitive impairment severe enough to impact daily living, and these four cases (13.3%) were observed in the subclinical hypothyroidism group (Table 6).

**Table 5:** PHQ-9 assessment of study participants. P-value <0.05 was considered significant. PHQ-9: Patient Health Questionnaire-9

Epworth category	Overall (n=81)	Subclinical hypothyroidism, n (%)	Overt hypothyroidism (%)	p-Value
Low normal daytime sleepiness	54	17 (56.7%)	37 (72.5%)	0.412
Normal daytime sleepiness	14	8 (26.7%)	6 (11.8%)	
Mild excessive daytime sleepiness	9	4 (13.3%)	5 (9.8%)	
Moderate excessive daytime sleepiness	1	0	1 (2%)	
Severe excessive daytime sleepiness	3	1 (3.3%)	2 (3.9%)	

**Table 6:** MoCA assessment of study participants. P-value <0.05 was considered significant. MoCA scale: Montreal Cognitive Assessment scale

MoCA	Overall n	Subclinical hypothyroidism, n (%)	Overt hypothyroidism, n (%)	p-Value
Normal	53	21 (70%)	32 (62.75%)	0.400
Mild cognitive impairment	17	4 (13.3%)	13 (25.5%)	
Moderate cognitive impairment	4	1 (3.3%)	3 (5.9%)	
Severe cognitive impairment	0	0	0	
Unable to perform	7	4 (13.3%)	3 (5.9%)	

The overall prevalence of poor sleep quality, sleep apnea, excessive daytime sleepiness, and cognitive impairment was nearly identical in both groups regardless of thyroid dysfunction (Table 7).

**Table 7:** High-risk category in each group. PSQI: Pittsburgh Sleep Quality Index; MoCA: Montreal Cognitive Assessment; PHQ-9: Patient Health Questionnaire-9

MoCA	Overall n	Subclinical hypothyroidism, n (%)	Overt hypothyroidism, n (%)	p-Value
Normal	53	21 (70%)	32 (62.75%)	0.400
Mild cognitive impairment	17	4 (13.3%)	13 (25.5%)	
Moderate cognitive impairment	4	1 (3.3%)	3 (5.9%)	
Severe cognitive impairment	0	0	0	
Unable to perform	7	4 (13.3%)	3 (5.9%)	

It was found that 61.1% of normal BMI (18.5-22.9) individuals had sleep disturbance as assessed by PSQI and 5.6% of normal BMI individuals were at high risk of sleep apnea as assessed by the Berlin Questionnaire. The MoCA assessment showed about 11.1% of normal BMI had severe anxiety. However, other scales showed no significant disturbance in normal BMI individuals.

## Discussion

Over the past decade, there has been a significant increase in thyroid dysfunction rates in India. While the exact reason remains unknown, some factors, such as obesity and the rise in autoimmune disorders, have been suggested as potential contributors [14,15]. In addition, various endocrine disruptors (EDD) have been shown to have direct and indirect effects on thyroid dysfunction [16].

In our study, most participants were females (79%), and the mean age group was 38.5±13 years. There was no observed variance in sleep quality indices concerning sleep dysfunction and thyroid dysfunction. Although the study highlighted the prevalence of sleep dysfunction among patients, it did not establish any correlation with the levels of TSH.

In a study by Song et al., the sleep quality assessment among the subclinical hypothyroid subjects by PSQI showed a higher occurrence of poor sleep quality in subclinical hypothyroidism (67%) [17]. In our study, it was found that sleep abnormalities ranged from 26.6% to 66.7% in the subclinical hypothyroidism population based on the questionnaires used. However, in accordance with our study, the sleep severity did not show any association with the level of TSH. However, this study had a predominantly male population but with a similar age group.

In the same study, the population's BMI was 23.69±3.37, while in the present study, it was 26.65±5.22. Although around 24% of our population was normal weight, among these 5.6% showed a high risk of sleep apnea, and 61.1% showed significant sleep disturbance. Though 24% of the population had normal BMI, the majority also showed sleep disturbance. Thus, overweight and obesity cannot be the main contributors to sleep disturbance.

Further, studies by Nazem et al. showed increased TSH levels in poor sleepers [18]. Several studies have also shown the involvement of the pituitary thyroid axis in sleep-deprived individuals [19]. Although present study had predominantly female population in comparison to other studies that had male predominance, the results were similar. Hence, gender may not be a determinant of sleep disturbance.

Berlin risk categorization showed a higher risk of OSA in females with higher collar circumference and higher neck height ratio, thereby implying upper airway obstruction may be an important cause of sleep disturbance in hypothyroid individuals [20]. Due to gender-specific fat distribution patterns, wherein females show predominately lower body distribution, any changes in neck circumference might significantly impact females compared to males [21].

The increased occurrence of sleep apnea in thyroid dysfunction is attributed to obesity, macroglossia, dysfunction in airway musculature, deposition of mucopolysaccharides in upper airway tissues, and decreased ventilatory control of musculature [22]. Certain studies have documented increased occurrence of OSA in subclinical hypothyroidism [23]. In our study, the occurrence of sleep apnea, as assessed by ESS, was almost the same in both overt and subclinical hypothyroidism. In the presence of comorbidities such as dyslipidemia or sleep apnea, as seen in the present study involving individuals with subclinical hypothyroidism, there are no definitive guidelines for treatment; however, some patients may benefit from it.

The exact mechanism by which thyroid dysfunction causes sleep disturbance is elusive. Certain studies have shown that insomnia is much more prevalent in the presence of medical comorbidities [24]. The symptoms of thyroid dysfunction, such as fatigue, constipation, and intolerance to cold, could account for insomnia in affected individuals. Further studies are needed to understand the underlying reasons for this phenomenon in individuals with subclinical hypothyroidism.

The study’s limitations include the subjective assessment of sleep dysfunction. As the study included predominantly female subjects generalization is less feasible. Further research is required regarding objective assessment, such as polysomnography, to assess and follow-up on sleep dysfunction following levothyroxine supplementation. To the best of our knowledge, not many studies have compared sleep dysfunction between subclinical and overt hypothyroidism which might be attributed to the strength of the study. Also, this study considers physical parameters along with the questionnaires to asses the severity of sleep dysfunction in individuals.

## Conclusions

Our study showed an equal occurrence of poor sleep quality in both subclinical (66.7%) and overt hypothyroidism (60.8%) patients. Further sleep apnea was higher in females with greater collar circumference and neck-height ratio. Other dysfunctions as assessed by Epworth Sleepiness Score, PHQ-9, and MOCA showed no significant difference between the subclinical and overt hypothyroidism group. This suggests that the severity of hypothyroidism did not correlate with the occurrence of underlying sleep disorders.

In conclusion, investigating the spectrum of sleep abnormalities in primary hypothyroidism patients holds significant potential for improving diagnosis, treatment strategies, and overall patient outcomes. This research can contribute to a more comprehensive understanding of the multifaceted relationship between thyroid dysfunction and sleep, ultimately leading to better patient care.

## Figures and Tables

**Figure 1:**
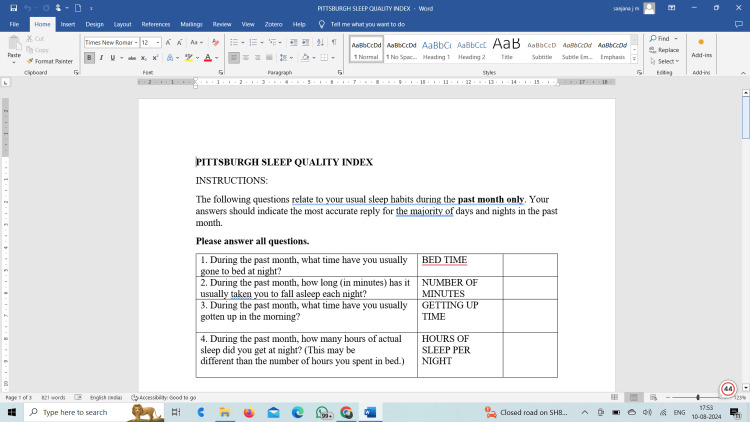
PSQI Questionnaire (page 1). PSQI: Pittsburgh Sleep Quality Index

**Figure 2:**
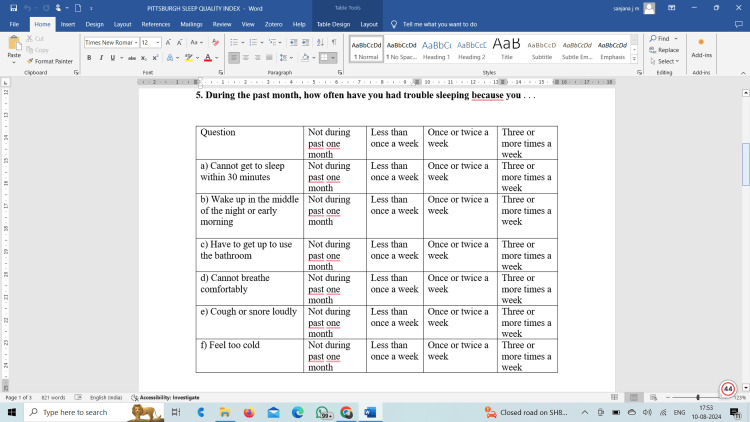
PSQI Questionnaire (page 2). PSQI: Pittsburgh Sleep Quality Index

**Figure 3:**
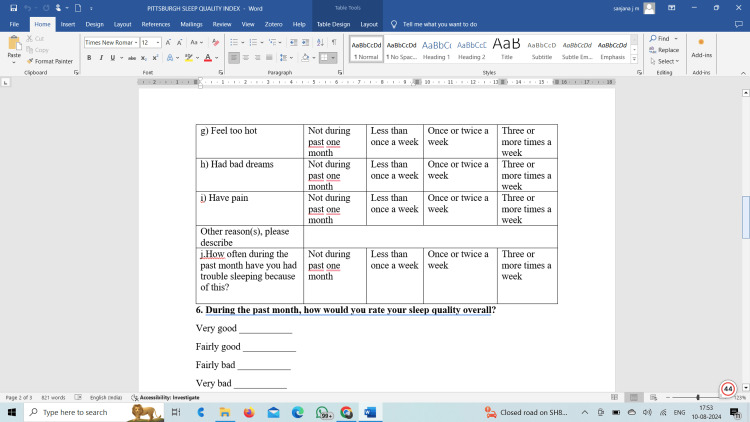
PSQI Questionnaire (page 3). PSQI: Pittsburgh Sleep Quality Index

**Figure 4:**
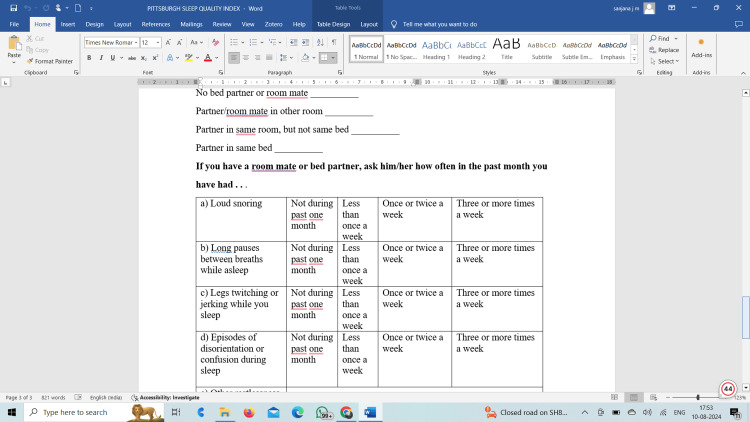
PSQI Questionnaire (page 4). PSQI: Pittsburgh Sleep Quality Index

**Figure 5:**
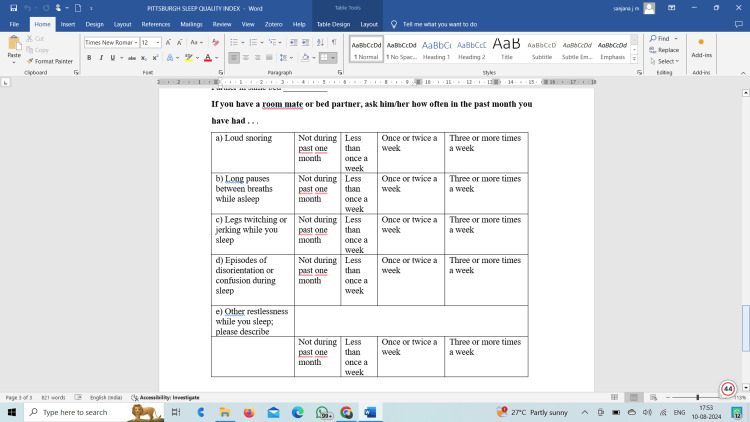
PSQI Questionnaire (page 4). PSQI: Pittsburgh Sleep Quality Index

**Figure 6:**
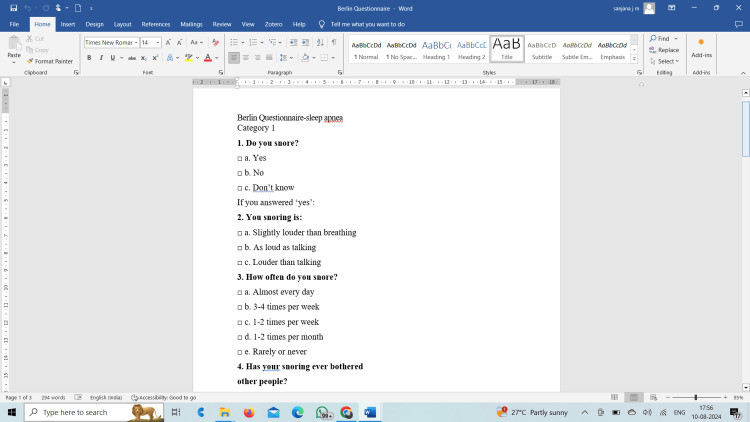
Berlin Questionnaire (page 1).

**Figure 7:**
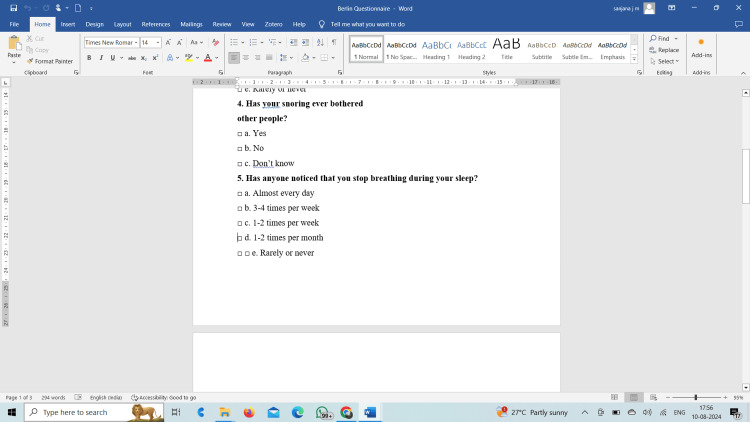
Berlin Questionnaire (page 2).

**Figure 8:**
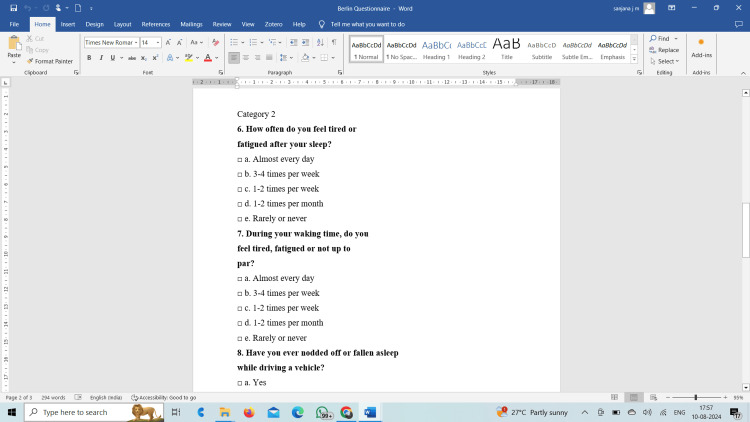
Berlin Questionnaire (page 3).

**Figure 9:**
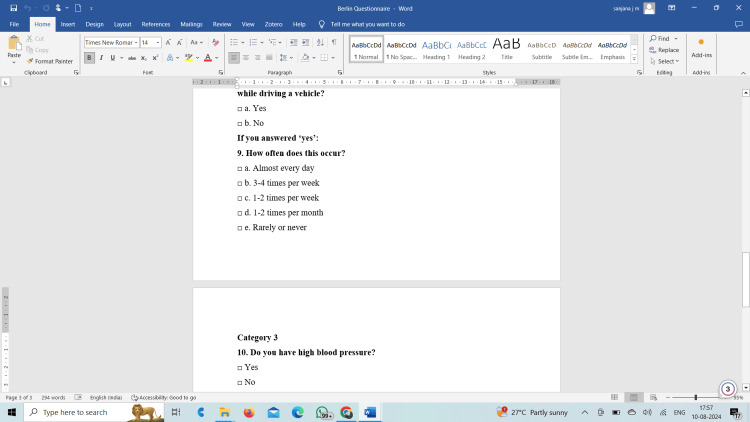
Berlin Questionnaire (page 4).

**Figure 10:**
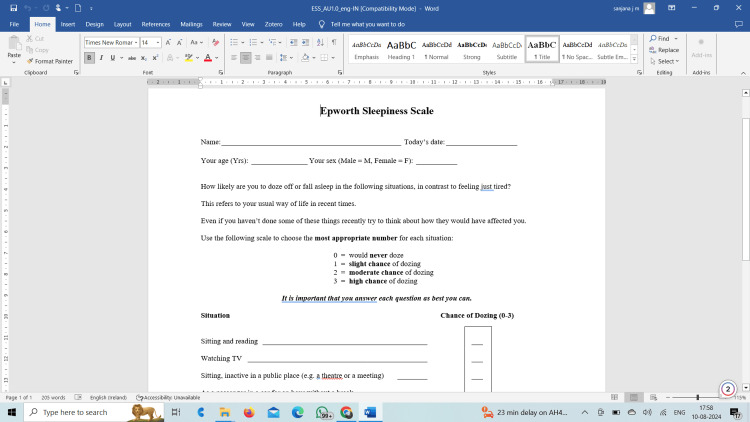
Epworth Sleepiness Scale (page 1).

**Figure 11:**
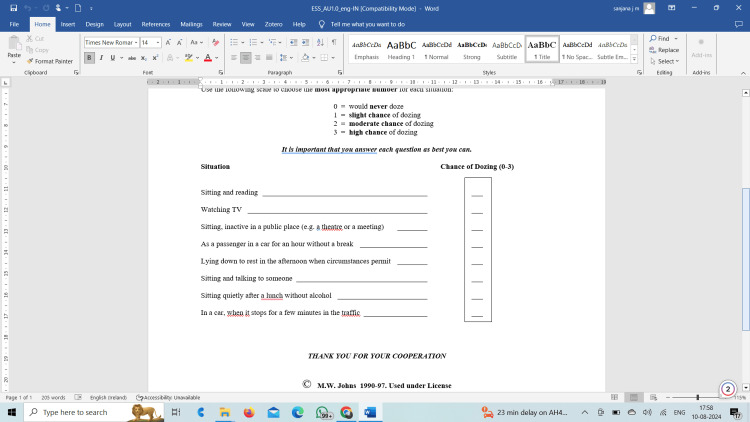
Epworth Sleepiness Scale (page 2).

**Figure 12:**
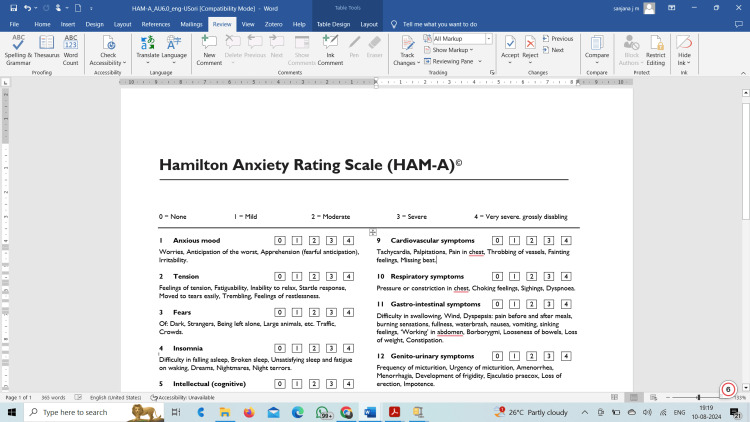
HAM-A Questionnaire (page 1). HAM-A: Hamilton Anxiety Rating Scale

**Figure 13:**
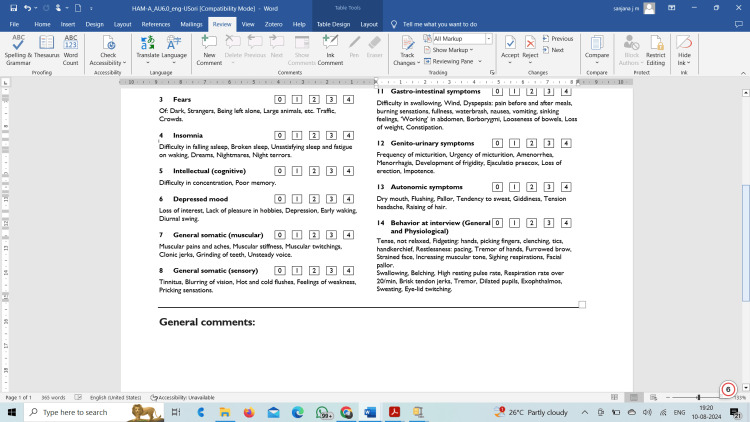
HAM-A Questionnaire (page 2). HAM-A: Hamilton Anxiety Rating Scale

**Figure 14:**
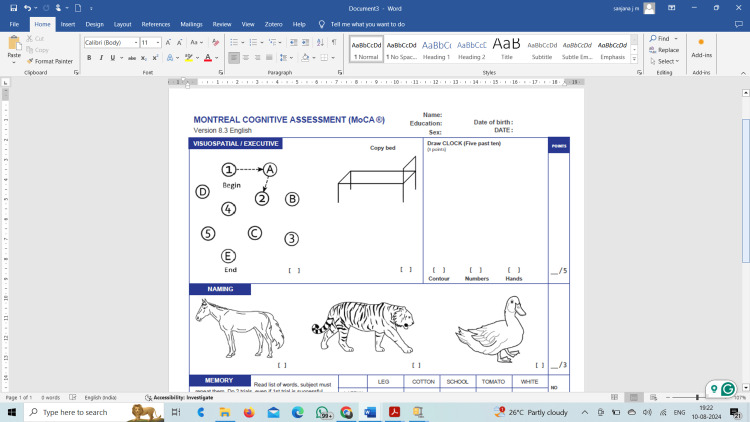
MoCA Questionnaire (page 1). MoCA: Montreal Cognitive Assessment

**Figure 15:**
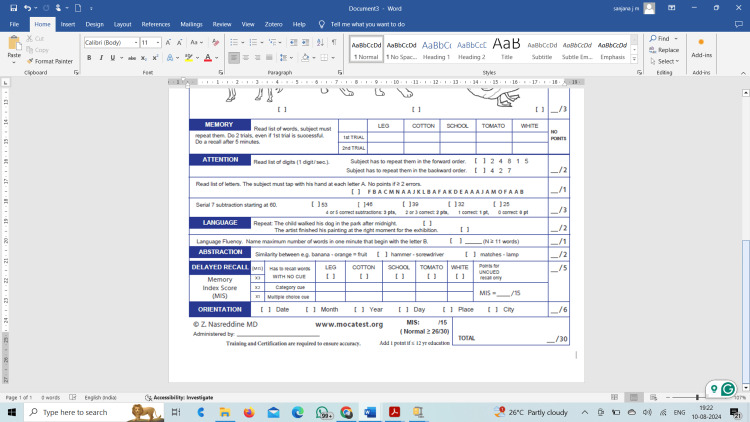
MoCA Questionnaire (page 2). MoCA: Montreal Cognitive Assessment

**Figure 16:**
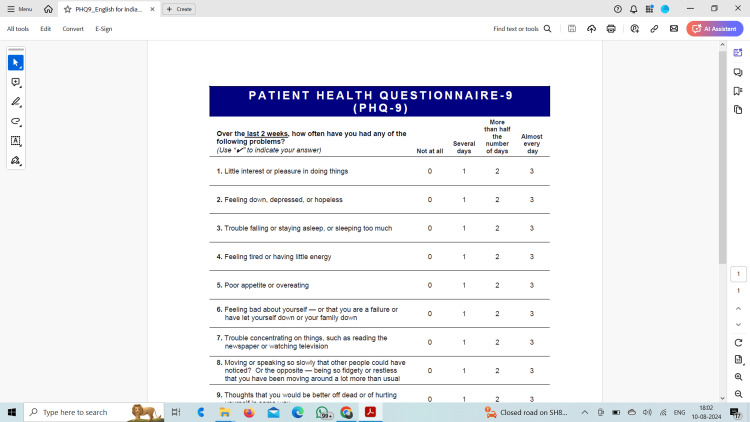
PHQ-9 Questionnaire (page 1). PHQ: Patient Health Questionnaire

**Figure 17:**
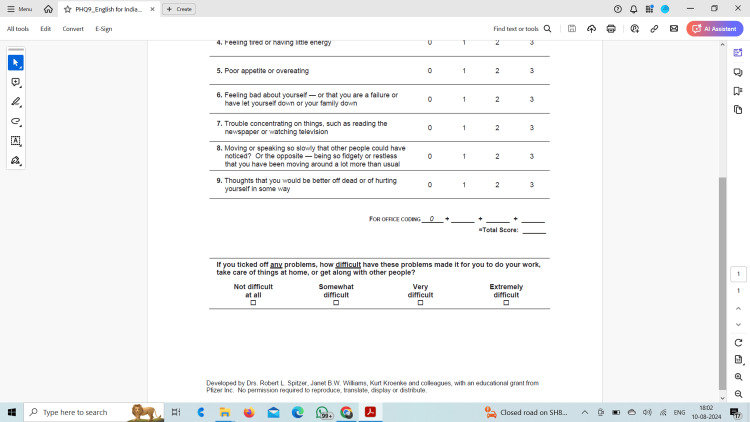
PHQ-9 Questionnaire (page 2). PHQ: Patient Health Questionnaire

## Appendices

Appendix 1

PSQI Questionnaire

The permission to use PSQI was obtained from Daniel J. Buysse, MD, distinguished professor of psychiatry, medicine, and clinical and translational science, UPMC Endowed Chair in Sleep Medicine University of Pittsburgh, School of Medicine.

Appendix 2

Berlin Questionnaire

The permission to use the Berlin Questionnaire was obtained from Prof. (h.c) Nikolaus Netzer MD PhD. Hermann Buhl Institute, University Innsbruck, Bad Aibling, Germany. Senior Researcher Institute for Mountain Emergency Medicine and Scientific Medical Director, Terra X Cube, Eurac Research, Bad Aibling, Germany.

Appendix 3

Epworth Sleepiness Scale

The permission to use ESS was obtained from MAPI Research Trust Lyon, France, no. 99581.

Appendix 4

HAM-A Questionnaire

The permission to use the HAM-A Questionnaire was obtained from MAPI Research Trust, Lyon, France, no. 99580.

Appendix 5

MoCA and PHQ-9

These questionnaires are available freely and hence used as free questionnaires.

Appendix 6
